# Cooperative breeding shapes post‐fledging survival in an Afrotropical forest bird

**DOI:** 10.1002/ece3.2744

**Published:** 2017-04-07

**Authors:** Dries Van de Loock, Diederik Strubbe, Liesbeth De Neve, Mwangi Githiru, Erik Matthysen, Luc Lens

**Affiliations:** ^1^ Terrestrial Ecology UnitGhent UniversityGhentBelgium; ^2^ Department of ZoologyNational Museums of KenyaNairobiKenya; ^3^ Evolutionary Ecology GroupUniversity of AntwerpCampus Drie EikenWilrijkBelgium; ^4^ Wildlife WorksVoiKenya

**Keywords:** group size, helpers, juvenile independence, postfledging mortality, radio‐telemetry

## Abstract

For avian group living to be evolutionary stable, multiple fitness benefits are expected. Yet, the difficulty of tracking fledglings, and thus estimating their survival rates, limits our knowledge on how such benefits may manifest postfledging. We radio‐tagged breeding females of the Afrotropical cooperatively breeding Placid greenbul (*Phyllastrephus placidus)* during nesting. Tracking these females after fledging permitted us to locate juvenile birds, their parents, and any helpers present and to build individual fledgling resighting datasets without incurring mortality costs or causing premature fledging due to handling or transmitter effects. A Bayesian framework was used to infer age‐specific mortality rates in relation to group size, fledging date, maternal condition, and nestling condition. Postfledging survival was positively related to group size, with fledglings raised in groups with four helpers showing nearly 30% higher survival until independence compared with pair‐only offspring, independent of fledging date, maternal condition or nestling condition. Our results demonstrate the importance of studying the early dependency period just after fledging when assessing presumed benefits of cooperative breeding. While studying small, mobile organisms after they leave the nest remains highly challenging, we argue that the telemetric approach proposed here may be a broadly applicable method to obtain unbiased estimates of postfledging survival.

## 1 Introduction

While it has generally been acknowledged that avian group living has to confer multiple benefits to be evolutionary stable (Dickinson & Hatchwell, 2004), the difficulty of following birds after fledging (reviewed by Cox, Thompson, Cox, & Faaborg, 2014) limits our knowledge on postfledging benefits. Yet, offspring of cooperative breeders typically receive extended care after fledging, suggesting that helpers can contribute substantially to a breeder's fitness during this phase too (Langen, 2000). Quantifying postfledging survival in group‐living species can hence result in more accurate measures of reproductive success and provide new insights into the ecology and evolution of sociality (Hatchwell et al., 2004; Preston, Briskie, & Hatchwell, 2016). However, studies addressing possible effects of cooperative breeding on postfledging survival have yielded mixed results (see Appendix S1 and Table S1), possibly due to methodological and/or taxonomic heterogeneity. For instance, few studies quantified survival during early dependency, when avian mortality rates are assumed to be highest (e.g., Sankamethawee, Gale, & Hardesty, 2009), and only some have carried out this in Afrotropical forest birds, where cooperative breeding is common (Jetz & Rubenstein, 2011). Most studies found that helpers appear to have a neutral effect on postfledging survival. Yet, the influence of helpers on postfledging juvenile survival may vary temporally. For example, helpers may positively influence survival in the first days after fledging but not later on (e.g., Williams & Hale, 2006; Mumme, Bowman, Pruett, & Fitzpatrick, 2015). If postfledging survival is only assessed at a longer time span, the impact of cooperative breeding on juvenile survival may be misunderstood (e.g., Covas, Deville, Doutrelant, Spottiswoode, & Grégoire, 2011).

Here, we use an innovative radio‐tracking approach to obtain postfledging survival estimates of an Afrotropical cooperatively breeding bird and relate these to group size. Although radio‐tagging nestlings may appear most straightforward, both premature fledging due to handling and detrimental transmitter effects can bias survival estimates (Mattsson, Meyers, & Cooper, 2006; Streby et al., 2013). To overcome this, we tagged breeding females prior to fledging and hence were able to track fledglings without the associated costs to juveniles. Age‐specific postfledging mortality rates were obtained by fitting parametric survival functions to resighting data using a Bayesian framework (Colchero, Jones, & Rebke, 2012).

## 2 Material and Methods

### 2.1 Study system

Between November 2014 and April 2015, resighting data were collected for 40 juveniles fledged from 25 Placid greenbul (*Phyllastrephus placidus*) nests. Nests were detected by experienced field assistants in five remnant cloud forest patches within the Taita Hills, southeast Kenya (1500 m A.S.L., 3°25′S, 38°20′E). The Taita Hills represent the northernmost range of the Eastern Arc Mountains biodiversity hotspot and are isolated from similar highland areas by low‐altitudinal savannah (900 m A.S.L.) in every direction (Lovett & Wasser, 1993; Myers, Mittermeier, Mittermeier, da Fonseca, & Kent, 2000). The need for agricultural land strongly pressured the indigenous cloud forest cover since precolonial times, and current distribution resembles an archipelago imbedded within small agroforestry fields and surrounded by exotic plantations (Pellikka, Lötjönen, Siljander, & Lens, 2009).

The Placid greenbul is a medium‐sized insectivorous passerine of montane cloud forests understory. They build open cup‐shaped nests at the forks of saplings, shrubs or climbers that resemble trapped leaf debris. Clutches (2–3 eggs) are incubated by the female, hatch after about 15 days, and fledge 11 (10–13) days later. In Taita Hills, stable territories with ill‐defined borders are defended by pairs or cooperatively breeding groups consisting of a socially monogamous breeding pair and up to four helpers (Van de Loock, D. unpubl. data). Breeding coincides predominantly with the onset of the short rains in November and can last until March. Predation pressure is high with up to 70% of initiated nests failing completely (Spanhove, Callens, Hallmann, Pellikka, & Lens, 2014). Importantly, an intensive color‐banding effort is ongoing since 1996 through standard‐effort ringing and nest monitoring, with at least 70% of the population being individually recognizable at all times (Lens, L. unpubl. data).

### 2.2 Resighting data

To relocate fledglings, we attached lightweight VHF transmitters (Pip tag, Biotrack Ltd., Wareham, U.K.; <4% body mass) to breeding females during nesting, using a leg‐loop harness (Rappole & Tipton, 1990). There were no indications that maternal nest attentiveness and mobility were affected by tagging. To distinguish between individuals, nestlings were banded with a metal ring and a unique combination of color rings prior to fledging. Each mother was relocated within 6 days after fledging of her brood and every subsequent fifth or sixth day until 55 days postfledging, when fledglings are considered nutritionally independent (pers. obs. Van de Loock, D). Upon location of the tagged female, known offspring were intensively searched for over a 45‐min search session. Flocks forage as coherent groups, and known flock members could nearly always be observed within this timeframe (Van de Loock, D. pers. obs.), hence suggesting that repeatedly undetected offspring was almost certainly dead. In addition, capture–recapture data since 2007 revealed no juvenile dispersal within 2 months after fledging (Lens, L. unpubl. data). Therefore, disappearance due to dispersal instead of mortality and therefore false‐negative observations (i.e., offspring considered deceased while still alive) were highly unlikely.

### 2.3 Statistical modeling

#### 2.3.1 Predictor variables

We considered four variables as predictors of postfledging survival. (i) G*roup size* (GS). GS was determined through multiple focal observations around the nest at several occasions pre‐ and posthatching, combined with targeted mistnet traps with tape lure after hatching (average nestling age at targeted mistnet traps: 6.3 days, range 4–10, *n* = 37) and video recording during feeding of nestling (average nestling age at recording: 8 days, range 6–10, *n* = 28). The tape lure track consisted of recorded distress calls of conspecifics, intermitted with silence periods, and was deployed for a maximum duration of 10 min. Allofeeding behavior was recorded from 7 a.m.–1 p.m. continuously using a HD video camera set up at approximately 1.5 m from the nest. *P. placidus* individuals are known to be very sedentary and are often found in the same small area for long consecutive periods (Fry & Keith, 2000). Long‐term observations of groups with known composition in our study area confirmed that both their size and composition rarely change during pre‐ and postfledging periods (Van de Loock, D. unpubl. data). Group size is the combined sum of the breeding pair and all helpers and was distributed as follows: 2: 6 broods; 3: 8 broods; 4: 6 broods; 5: 4 broods; and 6: 1 brood. When a breeding pair is thus not aided by helpers, the GS is 2, while in a GS of 6, four helpers aid the breeding pair. For a subset of fledglings (*n* = 28), we could reliably determine the number of allofeeders (AF) as well. Numbers of allofeeders were distributed as follows 1: 2 broods; 2: 11 broods; 3: 3 broods; and 4: 1 broods. As with GS, AF is the combined total of the breeding pair and the helpers. Only the male or female breeder provided the nestling with care when AF was 1, while both breeders received aid from 2 helpers when AF was 4. GS was related to AF (r = .46; *p* = .01; *n* = 28); (ii) *Scaled Mass Index* (SMI). Nestlings were measured and weighed at an average age of 9 days (range 7–11 days), and SMI values were calculated that accounted for age and variation in body size (following (Peig & Green, 2009)). SMI was not related to GS, AF or brood size (all *p* > .5); (iii) *Fledging date* (FLD). This was calculated as the number of days since the earliest fledged nest. FLD was not related to GS (GLM with Poisson errors, χ^2^_(26)_ = 543.16; *p* = .34); and (iv) *Maternal condition* (MC). This was inferred from daily feather growth rates of the breeding female, quantified by the width of five consecutive growth bars averaged over the left and right second outermost rectrix (ptilochronology sensu (Grubb, 2006)) and reflecting individual and environmental quality during the period of feather growth (Grubb, 2006). To correct for variation in body size, residuals from an ordinary least squares regression of average bar width against tarsus were used (Vangestel, Braeckman, Matheve, & Lens, 2010). MC was not related to GS (LM, F_4,19_  = 0.45; *p* = .77).

#### 2.3.2 Bayesian survival trajectory analysis

Time‐specific mortality rates were estimated using the Bayesian Survival Trajectory Analysis (BaSTA) package in R (Colchero et al., 2012). This package is specifically designed for right‐censored data (i.e., deaths unknown) and allows for obscured timing of death resulting from our indirect telemetry approach. First, the most appropriate mortality function was chosen based on the lowest deviance information criterion (DIC), after comparing all possible functions (Gompertz, Exponential, Logistic, and Weibull) and shapes (Simple, Weibull, Bathtub) (Millar, 2009). Second, the most appropriate model was used to infer the relationship between our four predictor variables and postfledging mortality. Forest patch ID in which the nest was located was added as a covariate in each model (Colchero et al., 2012). Models were initially run with all four predictor variables included (i.e., group size, scaled mass index, fledging date, maternal condition). Nonrelevant variables (i.e., 95% confidence interval of the model estimate gamma [γ] includes 0) were removed in a stepwise manner until only relevant models remained (“minimal adequate model,” MAM) (Klein & Moeschberger, 2003). Reported parameter values were derived from the MAM for the relevant variables, and parameter values of nonrelevant variables were obtained by forcing the variable into the MAM. Models were run 100 times, and outputs were averaged to obtain stable estimates. Finally, as the BaSTA analytical framework does not accommodate random effects, we bootstrapped the final model 100 times while only selecting one fledgling per nest. Mortality functions were optimized using a Markov Chain Monte Carlo (MCMC) simulation procedure using four parallel simulations with 20,000 iterations, 2001 burn‐in periods, and 150 interval sampling each. Models converged appropriately, and serial autocorrelation or choice of priors did not affect the model estimation. All statistical analyses were run in R 3.2.2 (R Core team, 2015).

## 3 Results

**Figure 1:**
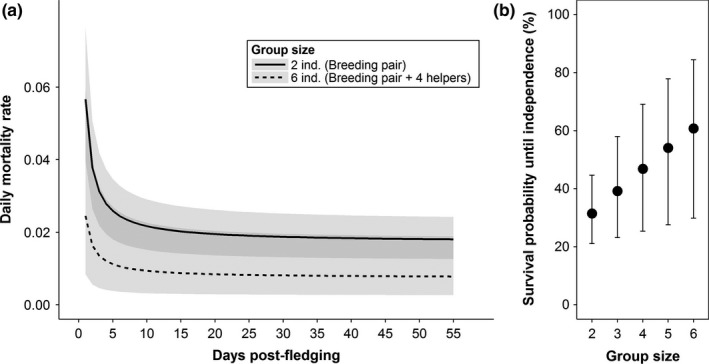
Estimates of (a) daily mortality rates during postfledging dependency for juveniles raised by pairs (group size = 2) or by the largest recorded group (group size = 6), and (b) survival probability until independence in relation to group size. Shaded area and whiskers reflect 95% CI in (a) and (b), respectively

Overall, fledglings had a 45.2% survival probability up to nutritional independence (95% CI 15.7%–70.1%). The postfledging mortality rate was best explained by a Weibull function with a Makeham shape (∆DIC second best model: 13.9) and decreased over time. Fledglings in larger groups and with a better body condition had lower mortality rates (Table 1, Figure 1a). Fledglings in groups with four helpers had a 29.3% higher survival up to independence compared with individuals raised by a breeding pair only (Figure 1b), while the heaviest fledglings had 8.2% higher survival compared with the lightest ones. Both factors did not significantly interact, and mortality was not affected by fledging date or maternal condition (Table 1). In the subset of nests for which the number of allofeeders could be reliably quantified, the latter failed to predict postfledging survival when substituting GS in the MAM (average posterior: γ AF = −0.19, 95% CI −0.74–0.34), while SMI still did (average posterior: γ SMI = −0.20, 95% CI −0.28 to −0.12). Finally, the bootstrapping procedure rerunning the MAM randomly selecting one fledgling per nest produced similar estimates, so we consider our analysis unbiased with respect to nonindependence of fledglings.

**Table 1:** Posterior estimates of gamma (γ) for each covariate and interaction

	Estimate	SE_estimate_	Lower 95% CI	Upper 95% CI
Group size	−0.41	0.17	−0.74	−0.092^a^
Scaled mass index	−0.14	0.038	−0.21	−0.061^a^
Ordinal fledging day	−0.016	0.0086	−0.033	0.00084
Maternal condition	−0.18	0.17	−0.51	0.13
Group size a scaled mass index	−0.15	0.61	−1.28	1.08

a Relevant predictor of fledgling mortality; sign of estimate indicates direction of relationship.

## 4 Discussion

By estimating daily mortality rates of fledglings based on telemetry of mothers during dependency, we show that group size had a positive effect on postfledging survival, independently of nestling condition. The few studies that quantified helper effects on postfledging survival yielded mixed results (Table S1). However, these studies strongly varied in timing over which postfledging survival was quantified, from 2 weeks (e.g., Mumme et al., 2015) to 1 year (e.g., Covas et al., 2011). As selection pressures can vary temporally, presence of helpers may enhance fledging survival soon after fledging but not later on (e.g., Mumme et al., 2015), for example, due to local competition between helpers and fledglings (Covas et al., 2011). For example, the number of juvenile Brown jays (*Cyanocorax morio*) surviving to 30 days after fledging was strongly correlated with group size, while the number of offspring still alive after 1 year showed only a weak relationship (Williams & Hale, 2006). Ignoring temporal dynamics of helper contributions to fledgling survival can thus obscure subtle mechanisms through which cooperative breeding impacts on species' life‐history traits.

Apart from duration over which survival rates are being assessed, helper effects may also be confounded by territory quality, as better territories often hold higher‐quality breeding pairs, larger groups or both (Eguchi, Yamagishi, Asai, Nagata, & Hino, 2002). While such intertwined effects are best disentangled by removal or cross‐foster experiments (Brown, Brown, Brown, & Dow, 1982), the latter potentially disrupt social relationships within the group (Mumme, 1992) and may be too intrusive for tropical species with a slow life history and high disturbance sensitivity. Nonetheless, our correlative approach shows group benefits independently of maternal condition.

How exactly helpers contribute to the reduced mortality in *P. placidus* fledglings currently remains unknown. Both antipredator behavior and alloparental care have been suggested as important, nonexclusive direct mechanisms (Mcgowan & Woolfenden, 1990; Ridley, 2007). In addition, helpers may also contribute to the learning of foraging skills, although empirical studies on this mechanism remain scarce (Heinsohn, 1991; Langen, 1996). Based on our ecological knowledge of *P. placidus*, we hypothesize that antipredator behavioral strategies may underlie the positive effect of helpers on postfledging survival observed in our study population. Indeed, while nest‐predation rates were already shown to be high in *P. placidus* (Spanhove et al., 2014), a recent pilot study in which transmitters were placed on *P. placidus* nestlings showed that over 90% of postfledging mortality could be attributed to predation as well (Van de Loock, D. unpubl. data). The latter often involved African Goshawks (*Accipiter tachiro)*, an aerial predator also specialized on bird eggs and nestlings (Spanhove et al., 2014). As overall survival until independence (45.2%) is low in *P. placidus* compared with other tropical species (Lloyd & Martin, 2016; Sankamethawee et al., 2009), antipredator strategies hence are an obvious avenue for increasing fitness.

We acknowledge our data cannot disentangle the relative contributions of antipredator behavior versus alloparental care. Quantifying the role of alloparental care can be made through relating postfledging survival to feeding rates or the number of allofeeders in case not all group members allofeed the young (Ridley, 2007; Mcgowan & Woolfenden, 1990; Brouwer, Richardson, & Komdeur, 2012). For *P. placidus*, survival models run on a subset of data for which the number of allofeeders at the nest was known showed that fledgling survival was not correlated with number of allofeeders. Data on postfledging feeding events and on the identity of the allofeeder, however, remain scarce for *P. placidus*. The fact that we did not find any relationship should therefore be interpreted cautiously, as we currently cannot exclude the possibility that alloparental care contributes to postfledging survival as well.

In conclusion, we demonstrate that variation in group size in cooperative breeding species can substantially affect juvenile survival and thereby reproductive success. We agree with Heinsohn (1992) that full breeding cycle research in cooperative breeding ecology is crucial, yet remains rare due to logistic difficulties when studying evasive, mobile organisms during the early postfledging phase (Marra, Cohen, Loss, Rutter, & Tonra, 2015). We believe that the telemetric approach employed here may help to overcome some of these challenges and may ultimately lead to a better mechanistic understanding of relationships between cooperative breeding and postfledging survival.

## Data Accessibility

Data are available from the Dryad repository doi:10.5061/dryad.b4s20. Is a provisional DOI (submitted to Dryad on 11/01/2017).

## Conflict of Interest

None declared.
