# Supplementary material for: Cooperative breeding shapes post‐fledging survival in an Afrotropical forest bird
Source: Ecol Evol. 2017 Apr 7;7(10):3489–93. doi: 10.1002/ece3.2744 (PMC5433992; doi:10.1002/ece3.2744)
Supplement: Supplementary file 1 [file ECE3-7-3489-s001.docx]

**Electronic Supporting Information**

Van de Loock *et al.* “Cooperative breeding shapes post-fledging survival in an Afrotropical forest bird”

**APPENDIX S1**

*Literature review*

A literature review on the effects of helpers on post-fledging survival in altricial cooperatively breeding birds yielded a limited number of studies. Three approaches were used: (i) a systematic search in Web of Science using a combination of specific keywords (post-fledging, post-fledgling, postfledging, juvenile survival, or fledgling survival and cooperative bird). (ii) a systematic search for specific cooperative breeding species listed in review papers (Kingma et al. 2010), complemented with additional species noted in the appendix of (Li et al. 2015) and in (Cockburn 1998; Hatchwell 1999; Legge 2000; Khan & Walters 2002) using Web of Science (keywords : Latin name and English name in combination with keywords fledgling, juvenile, postfledging or post-fledging). When multiple studies have been conducted on the same species, all are incorporated if methodology or outcome differs. Otherwise the one with the highest sample size is retained.

**TABLE S1**. Overview of key methods and results of case-studies on effects of helpers on post-fledging survival in altricial cooperatively breeding birds.

| **Species** | **Ecoregion ^a^** |  | **Methodology** | | |  | **Helper effect on survival ^e^** |  | **Reference** |
| --- | --- | --- | --- | --- | --- | --- | --- | --- | --- |
|  |  |  | **Follow-up period (days) ^b^** | **Detection Method ^c^** | **Statistical Method ^d^** |  |  |  |  |
| Florida Scrub Jay (*Aphelocoma coerulescens)* | NA (5) |  | 60 _(ND)_ | O | T |  | **POS**_Cat_ |  | (Mumme 1992) |
|  |  |  | 10 | O, MT | T |  | **POS**_Cont_ |  | (Mumme et al. 2015) |
|  |  |  | 10 – 70 _(ND)_ * | O, MT | T |  | **NEU**_Cont_ |  | (Mumme et al. 2015) |
| Arabian Babblers (*Turdoides squamiceps)* | PA (13) |  | 56 _(ND)_ | O | T |  | **NEU**_Cont_ / **POS** * |  | (Ridley 2007) |
|  |  |  | 365 | O | T |  | **NEU**_Cont_ / **POS** * |  | (Ridley 2007) |
| Ground Tit (*Pseudopodoces humilis)* | PA (10) |  | 365 | MT | T |  | **POS**_Cat_ * |  | (Li et al. 2015) |
| Rifleman (*Acanthisitta chloris)* | AA (4) |  | 365 | O | T |  | **NEU**_Cat_ |  | (Sherley 1990) |
|  |  |  | 365 | O | T |  | **POS**_Cat_ * |  | (Preston et al. 2016) |
| Long-tailed Tit (*Aegithalos caudatus)* | PA (4) |  | 365 + | O | CMR_Yearly_ |  | **POS**_Cat_ |  | (McGowan et al. 2003) |
| Seychelles Warbler (*Acrocephalus sechellensis)* | AT (1) |  | 365 + | O, MT | CMR_Yearly_ |  | **NEU**_Cont_ / **POS**_Cont_ |  | (Brouwer et al. 2012) |
| Brown Jay (*Cyanocorax morio*) | NT (1) |  | 30 | O | T |  | **POS**_Cont_ |  | (Williams & Hale 2006) |
|  |  |  | 365 |  | T |  | **NEU**_Cont_**(POS**_Cat_) * |  | (Williams & Hale 2006) |
| Karoo Scrub Robin (*Cercotrichas coryphoeus)* | AT (12) |  | 21 | O | T |  | **NEU**_Cat_ |  | (Lloyd et al. 2009) |
| Puff-throated Bulbul (*Alophoixus pallidus)* | IM (2) |  | 56 _(ND)_ | O | CMR_Weekly_ |  | **NEU**_Cat_ |  | (Sankamethawee et al. 2009) |
| White-winged Chough (*Corcorax melanorhamphos)* | AA (4) |  | 30 * | O, MT | T |  | **NEU**_Cont_ |  | (Heinsohn 1992) |
|  |  |  | 365 | O | T |  | **NEU**_Cont_ |  | (Heinsohn 1992) |
| American Crow (*Corvus brachyrhynchos)* | NA (12) |  | 14 | O | T |  | **NEU**_Cat_ |  | (Caffrey 2000) |
|  |  |  | 60 | O | T |  | **NEU**_Cat_ |  | (Caffrey 2000) |
| Carrion Crow (*Corvus corone)* | PA (12) |  | ± 90 _(ND)_ | O | T |  | **NEU**_Cont_ |  | (Canestrari et al. 2011) |
|  |  |  | 45 _(ND)_ | RT | T |  | **NEU**_Cat_ |  | (Roldán et al. 2013) |
|  |  |  | 365 | RT | T |  | **NEU**_Cat_ |  | (Roldán et al. 2013) |
| White-fronted Bee-eater (*Merops bullockoides)* | AT (7) |  | 180 _(ND)_ | O | T |  | **NEU**_Cont_ |  | (Emlen & Wrege 1991) |
| White-browed Sparrow-weaver (*Plocepasser mahali)* | AT (7) |  | 180 | O, MT | T |  | **NEU**_Cont_ |  | (Lewis 1982) |
| White-throated Magpie-jay (*Calocitta formosa)* | NT (2) |  | 180 _(ND)_ | O | T |  | **NEU**_Cat_ |  | (Langen & Vehrencamp 1999) |
| Apostlebirds (*Struthidea cinerea)* | AA (8) |  | 365 | O, MT | T |  | **NEU**_Cont_ |  | (Woxvold & Magrath 2005) |
| Galapagos Mockingbird (*Nesomimus parvulus)* | NT (13) |  | 365 | O, MT | T |  | **NEU**_Cat_ |  | (Kinnaird & Grant 1982) |
| Splendid Fairywen (*Malurus splendens)* | AA (12) |  | 365 | O | T |  | **NEU**_Cat_ |  | (Russell & Rowley 1988) |
| Pygmy Nuthatch (*Sitta pygmaea)* | NA (5) |  | 365 | O, MT | T |  | **NEU**_Cat_ |  | (Sydeman et al. 1988) |
| Western Bluebird (*Sialia sialis)* | NA (12) |  | 365 | O | T |  | **NEU**_Cat_ |  | (Dickinson et al. 1996) |
| White-breasted Robin (*Eopsaltria georgiana)* | AA (12) |  | ± 49 _(ND)_ | O, MT | T |  | **NEU**_Cat_ |  | (Russell et al. 2004) |
|  |  |  | 365 | O, MT | T |  | **NEU**_Cat_ |  | (Russell et al. 2004) |
| Southern Pied Babbler (*Turdoides bicolor)* | AA (13) |  | 365 | O | T |  | **NEU**_Cont_ |  | (Ridley & Raihani 2008) |
| Sociable Weaver (*Philetairus socius)* | AT (13) |  | 365 + | T | CMR_Yearly_ |  | **NEG**_Cat_ |  | (Covas et al. 2011) |

^a^ Realm and biome (in brackets) sensu (Olson et al. 2001)
^b^ Since fledging, or noted if otherwise. Recalculated to days using 7 days in a week, 30 in a month and 365 in a year. ND : period coincides with nutritional dependency
^c^ O : targeted observation or study population censussing; MT : mist-net traps; RT : radio-telemetry
^d^ T : effect of helpers on post-fledging survival assessed using temporal invariable statistical methods (ex. regression analysis, GLM(M), contingency tables); CMR : capture-mark recapture/resighting analysis incorporates temporal variability (timeframe for parameter estimation in subscript)
^e^ POS, NEU & NEG : positive, neutral or negative effect of helpers on post-fledging survival. Cont and Cat : number of helpers analysed continuously or categorical. When effect of number of allofeeders on survival differs this is indicated using a forward slash ( / ).
* see references for study-specific details

**REFERENCES**

Brouwer, L., Richardson, D.S. & Komdeur, J., 2012. Helpers at the nest improve late-life offspring performance: evidence from a long-term study and a cross-foster experiment. *PloS one*, 7(4), p.e33167.

Caffrey, C., 2000. Correlates of reproductive success in cooperatively breeding western American crows: if helpers help, it’s not by much. *The Condor*, 102(August), pp.333–341.

Canestrari, D., Marcos, J.M. & Baglione, V., 2011. Helpers at the nest compensate for reduced maternal investment in egg size in Carrion Crows. *Journal of evolutionary biology*, 24(9), pp.1870–8.

Covas, R., Deville, A.S., Doutrelant, C., Spottiswoode, C.N. & Grégoire, A., 2011. The effect of helpers on the postfledging period in a cooperatively breeding bird, the Sociable Weaver. *Animal Behaviour*, 81(1), pp.121–126.

Dickinson, J.L., Koenig, W.D. & Pitelka, F.A., 1996. Fitness consequences of helping behavior in the Western bluebird. *Behavioral Ecology*, 7(2), pp.168–177.

Emlen, S.T. & Wrege, P.H., 1991. Breeding Biology of White-Fronted Bee-Eaters at Nakuru: The Influence of Helpers on Breeder Fitness. *The Journal of Animal Ecology*, 60(1), pp.309–326.

Heinsohn, R.G., 1992. Cooperative enhancement of reproductive success in White-Winged Choughs. *Evolutionary Ecology*, 6(2), pp.97–114.

Kinnaird, M.F. & Grant, P.R., 1982. Cooperative breeding by the Galapagos Mockingbird, *Nesomimus parvulus*. *Behavioral Ecology and Sociobiology*, 10(1), pp.65–73.

Langen, T.A. & Vehrencamp, S.L., 1999. How White-Throated Magpie-Jay helpers contribute during breeding. *The Auk*, 116(1), pp.131–140.

Lewis, D.M., 1982. Cooperative breeding in a population of White-browed Sparrow Weavers *Plocepasser mahali*. *Ibis*, 124, pp.511–522.

Li, Y., Li, S., Guo, C., Zhang, G., Zhou, Y. & Lu, X., 2015. Nest helpers improve parental survival but not offspring production in a high-elevation passerine , the Ground Tit *Pseudopodoces humilis*. *Ibis*, 157, pp.567–574.

Lloyd, P., Andrew Taylor, W., Du Plessis, M.A. & Martin, T.E., 2009. Females increase reproductive investment in response to helper-mediated improvements in allo-feeding, nest survival, nestling provisioning and post-fledging survival in the Karoo scrub-robin *Cercotrichas coryphaeus*. *Journal of Avian Biology*, 40(4), pp.400–411.

McGowan, A., Hatchwell, B.J. & Woodburn, R.J.W., 2003. The effect of helping behaviour on the survival of juvenile and adult long-tailed tits *Aegithalos caudatus*. *Journal of Animal Ecology*, 72(3), pp.491–499.

Mumme, R.L., 1992. Do helpers increase reproductive success - an experimental-analysis in the Florida scrub jay. *Behavioral Ecology and Sociobiology*, 31(5), pp.319–328.

Mumme, R.L., Bowman, R., Pruett, M.S. & Fitzpatrick, J.W., 2015. Natal territory size, group size, and body mass affect lifetime fitness in the cooperatively breeding Florida Scrub-Jay. *The Auk*, 132(3), pp.634–646.

Olson, D.M., Dinerstein, E., Wikramanayake, E.D., Burgess, N.D., Powell, G.V.N., Underwood, E.C., D’amico, J. a., Itoua, I., Strand, H.E., Morrison, J.C., Loucks, C.J., Allnutt, T.F., Ricketts, T.H., Kura, Y., Lamoreux, J.F., Wettengel, W.W., Hedao, P. & Kassem, K.R., 2001. Terrestrial ecoregions of the world: a new map of life on earth. *BioScience*, 51(11), p.933.

Preston, S.A.J., Briskie, J. V & Hatchwell, B.J., 2016. Adult helpers increase the recruitment of closely related offspring in the cooperatively breeding rifleman. *Behavioral Ecology*, p.Advance online publication.

Ridley, A.R., 2007. Factors affecting offspring survival and development in a cooperative bird: social, maternal and environmental effects. *Journal of Animal Ecology*, 76(4), pp.750–760.

Ridley, A.R. & Raihani, N.J., 2008. Task partitioning increases reproductive output in a cooperative bird. *Behavioral Ecology*, 19(6), pp.1136–1142.

Roldán, M., Martín-Gálvez, D., Rodríguez, J. & Soler, M., 2013. Breeding biology and fledgling survival in a Carrion Crow *Corvus corone* population of Southern Spain: a comparison of group and pair breeder. *Acta Ornithologica*, 48(2), pp.221–235.

Russell, E. & Rowley, I., 1988. Helper contributions to reproductive success in the Splendid Fairy-wren (*Malurus splendens*). *Behavioral Ecology and Sociobiology*, 22(2), pp.131–140.

Russell, E.M., Brown, R.J. & Brown, M.N., 2004. Life history of the White-breasted robin, *Eopsaltria georgiana* (Petroicidae), in south-western Australia. *Australian Journal of Zoology*, 52(2), pp.111–145.

Sankamethawee, W., Gale, G.A. & Hardesty, B.D., 2009. Post-Fledgling survival of the cooperatively breeding Puff-Throated Bulbul (*Alophoixus pallidus*). *The Condor*, 111(4), pp.675–683.

Sherley, G.H., 1990. Co-operative breeding in Riflemen (*Acanthissitta chloris*) benefits to parents , offspring and helpers. *Behaviour*, 112(1), pp.1–22.

Sydeman et al., W.J., 1988. Annual reproductive yield in the cooperative Pygmy Nuthatch (*Sitta pygmaea*). *Auk*, 105(1), pp.70–77.

Williams, D.A. & Hale, A.M., 2006. Helper Effects on Offspring Production in Cooperatively Breeding Brown Jays (Cyanocorax Morio). *The Auk*, 123(3), p.847.

Woxvold, I.A. & Magrath, M.J.L., 2005. Helping enhances multiple components of reproductive success in the cooperatively breeding apostlebird. *Journal of Animal Ecology*, 74(6), pp.1039–1050.
